# Heart rate variability as a marker of recovery from critical illness in children

**DOI:** 10.1371/journal.pone.0215930

**Published:** 2019-05-17

**Authors:** Lauren E. Marsillio, Tomas Manghi, Michael S. Carroll, Lauren C. Balmert, Mark S. Wainwright

**Affiliations:** 1 Division of Critical Care Medicine, Department of Pediatrics, Ann & Robert H. Lurie Children’s Hospital of Chicago, Chicago, IL, United States of America; 2 Department of Pediatrics, Yale School of Medicine, New Haven, CT, United States of America; 3 Data Analytics and Reporting, Ann & Robert H. Lurie Children’s Hospital of Chicago, Chicago, IL, United States of America; 4 Department of Preventive Medicine, Northwestern University Feinberg School of Medicine, Chicago, IL, United States of America; 5 Division of Pediatric Neurology, Department of Neurology, University of Washington, Seattle, WA, United States of America; University of Notre Dame Australia, AUSTRALIA

## Abstract

**Objectives:**

The purpose of this study was to Identify whether changes in heart rate variability (HRV) could be detected as critical illness resolves by comparing HRV from the time of pediatric intensive care unit (PICU) admission with HRV immediately prior to discharge. We also sought to demonstrate that HRV derived from electrocardiogram (ECG) data from bedside monitors can be calculated in critically-ill children using a real-time, streaming analytics platform.

**Methods:**

This was a retrospective, observational pilot study of 17 children aged 0 to 18 years admitted to the PICU of a free-standing, academic children’s hospital. Three time-domain measures of HRV were calculated in real-time from bedside monitor ECG data and stored for analysis. Measures included: root mean square of successive differences between NN intervals (RMSSD), percent of successive NN interval differences above 50 ms (pNN50), and the standard deviation of NN intervals (SDNN).

**Results:**

HRV values calculated from the first and last 24 hours of PICU stay were analyzed. Mixed effects models demonstrated that all three measures of HRV were significantly lower during the first 24 hours compared to the last 24 hours of PICU admission (p<0.001 for all three measures). In models exploring the relationship between time from admission and log HRV values, the predicted average HRV remained consistently higher in the last 24 hours of PICU stay compared to the first 24 hours.

**Conclusion:**

HRV was significantly lower in the first 24 hours compared to the 24 hours preceding PICU discharge, after resolution of critical illness. This demonstrates that it is feasible to detect changes in HRV using an automated, streaming analytics platform. Continuous tracking of HRV may serve as a marker of recovery in critically ill children.

## Introduction

Balance between the sympathetic and parasympathetic branches of the autonomic nervous system (ANS) is essential to maintaining systemic homeostasis and responding effectively to external stressors. During critical illness, loss of normal autonomic function can occur as a result of inflammation and/or injury. [1–3], The resultant disruption in systemic homeostasis due to ANS dysregulation or dysfunction has been associated with organ dysfunction, increased illness severity, and poor outcomes.[4–8] The most common method of objectively assessing ANS dysregulation is through measurement of HRV, which reflects the normal, physiologic alterations in the intervals in time between consecutive heart beats that occur when there is balance of sympathetic and parasympathetic inputs on the electrical conduction system of the heart. [9]. Numerous studies in both children and adults identified that decreases in HRV precede clinical deterioration, reflect response to therapy, and are associated with poor outcomes in a variety of conditions. [10–17] Therefore, heart rate variability (HRV) may be useful as an indicator of clinical acuity in the pediatric intensive care unit (PICU). [5,9] However, translating these findings into actionable information, available in real-time to clinicians in critical care units has yet to be achieved.

Despite the potential applicability of HRV to critical care, HRV analysis is not a standard part of clinical practice. Currently, the main clinically-validated application of real-time tracking of HRV in pediatrics is confined to the neonatal intensive care unit (NICU) where an HRV-based early warning system predicts deterioration from infection, respiratory compromise, and neurologic injury in premature infants. [18–20] Widespread adoption of HRV analysis into other inpatient settings has been limited by difficulties in implementing automated, continuous analyses in real-time, reliance on electrocardiographic (ECG) signals from bedside monitors, management of artifact or non-sinus heart beats, and lack of specificity, as well as standardization of measurements in the literature. [21,22]

Therefore, we sought to determine whether we could create a system where HRV is calculated continuously, in real-time in the PICU while overcoming the described issues and to identify whether the HRV values generated from this real-time system are clinically meaningful. As a first step in this process, we performed a pilot study where we tested the hypothesis that HRV would increase with recovery from critical illness in pediatric patients admitted to the PICU.

## Methods

### Study population

This pilot study was approved by the Ann & Robert H. Lurie Children’s Hospital of Chicago Institutional Review Board with a waiver of informed consent. We performed a retrospective chart review and evaluation of HRV measures derived from stored ECG waveforms acquired from critically-ill children using a convenience sample of those admitted to the PICU from January 1 to April 30, 2015. Patients were included if they were less than 18 years of age and had a PICU length of stay (LOS) of ≥ 48 hours (as we required two non-overlapping time windows for comparison). Patients were excluded if they died during their PICU stay (as we wanted to capture changes in HRV as a patient’s physiologic state evolved from one of critical illness, such as at PICU admission, to one of improved health, such as at the time of PICU discharge), had an active “do not attempt resuscitation” (DNAR) order (for similar reasoning as we presumed patients with DNAR orders may not have demonstrated improvements in their health at PICU discharge), had a cardiac pacemaker (which eliminates ANS-determined HRV), a condition of pre-existing autonomic dysregulation or known cardiac arrhythmias (as we would be unlikely to be able to detect true HRV or improvements in HRV in these patients). Patients were also excluded if they had incomplete HRV data for their first and last 24 hours of PICU stay.

### Data collection and HRV calculation

Patient data extracted from the electronic health record (Epic; Epic Systems Corporation, Verona, WI) included demographic data, admission diagnoses, pre-existing and newly acquired medical conditions, exposure to medications known to affect the ANS including steroids, vasoactive infusions, sedatives, narcotics, alpha-2 agonists, anti-epileptic medications, and anti-hypertensive medications, as well as use of invasive mechanical ventilation and information on the duration of ventilation.

HRV was calculated on all PICU patients in real-time from the ECG waveforms obtained by BedMasterEx (ExcelMedical; Jupiter, FL) from the bedside monitors (Philips IntelliVue MP70) and stored on a secure internal research server for future use. This study included analysis of three widely-used time-domain measures that capture both short- and long-term HRV, using previously described methods and briefly described as follows (4): the root mean square of successive differences of RR (or NN) intervals (RMSSD) in ms, the % of successive NN interval differences over 50 ms (pNN50), and the standard deviation of NN intervals (SDNN) in ms. These measures were calculated previously, in real-time from the bedside monitor, for presumptive sinus beats over consecutive 5 minute intervals. At the time of acquisition from the bedside monitor, an RR-interval time series was created, and this was converted into our three time-domain measures of HRV using the software platform IBM InfoSphere Streams (International Business Machines Corp; Armonk, NY) by an external consulting group, CléMetric, Madison, WI. The calculated HRV values were stored and later analyzed. Artifact was managed using an automated recursive procedure for detection of outliers.

In order to determine the accuracy of the real-time system, we compared patient-level average HRV values obtained from this system from the first and last 24 hours of PICU admission to values obtained through analysis of raw R-R interval data using Kubios HRV Premium version 3.1.0 (Kubios Oy, Kuopio, Finland) a commercially available, validated software program for HRV analysis. [23]

HRV data were collected and calculated for the first and last 24 hours of PICU stay. These time periods were selected in order to compare a time during which patients were most likely to be critically ill (i.e., first 24 hours of PICU admission) to a time representing resolution of critical illness (i.e., last 24 hours of PICU admission).

### Statistical analysis

We assessed the strength of the association between the two sets of HRV values obtained from our system and Kubios via Pearson’s correlation coefficient estimates for each of the three time-domain measures in the first and the last 24 hours. HRV values were averaged in the first and last 24 hours to allow for comparison. Because two measures can be highly correlated, but have little agreement, we also used Bland-Altman plots to visually assess agreement between the two measures. [24]

Descriptive statistics summarized all patient demographics and clinical characteristics. To visually assess HRV measurements in the first and last 24 hours of PICU admission, scatter plots of the average log HRV measurement over time from admission as well as by time of day were plotted with local polynomial regression (LOESS) curves (nonparametric method for fitting a smooth curve to better visualize trends in data) overlaid on top.

Mixed effect models with a fixed effect for time period (first 24 hours of PICU admission vs last 24 hours of PICU admission) and a random subject effect, to account for correlations of measurements within patients, were considered for each of the three HRV measurements using all available 5 minute HRV values. Model assumptions were assessed and log transformations of outcomes were applied, as necessary. We further explored the relationship between time from admission and HRV measurements by including fixed effects for time from admission and the interaction between time and period. The interaction term allowed us to assess if the relationship between time and HRV measurement was different in the first vs. last 24 hours. We explored associations between HRV measurements and each demographic and clinical variables of interest, in turn, to determine potential confounders. Adjusted models considered variables deemed significant (alpha = 0.2) or known to be associated with HRV measurements.

We then explored the relationship between the average of patients’ 5 minute HRV measurements over the entire 24 hour periods (first and last 24 hours) as well as first 24 hours and last 24 hours separately, and length of stay measures using spearman correlation coefficients. All analyses assumed a two-sided type 1 error rate of 0.05, and no corrections were made for multiple hypothesis tests.

## Results

**Fig 1:**
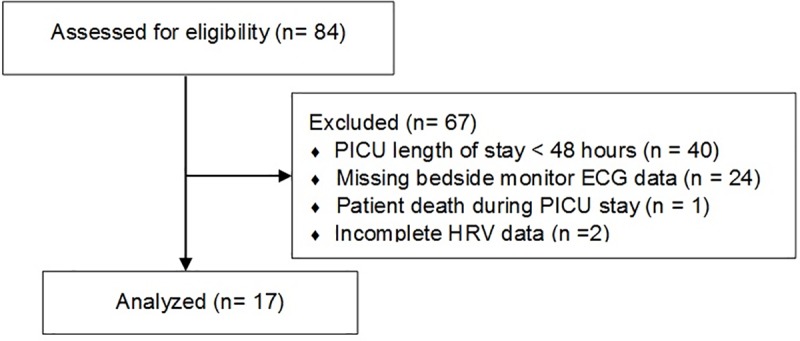
Cohort inclusion and exclusion flow diagram.

During the study period, a total of 84 patients were screened and 17 patients were included for analysis. (Fig 1) The majority of patients were male, were admitted with respiratory diagnoses, and had a median PICU length of stay (LOS) of 4 days (range of 2–18 days). (Table 1) Patient clinical and demographic data can be found in S1 Data.

**Table 1:** Patient demographic and clinical characteristics.

Variable	Median (Min-Max) / N (%)
**Age (years)**	1.21 (0.28–14.81)
**Hospital LOS (days)**	7 (2–46)
**PICU LOS (days)**	4 (2–18)
**Sex**	
Male	12 (70.6%)
Female	5 (29.4%)
**PICU Diagnosis Code**	
Respiratory	12 (70.6%)
Cardiac	1 (5.9%)
Post-surgical	1 (5.9%)
Other	2 (11.8%)
Sepsis/suspicion of sepsis	1 (5.9%)
**Ventilator use**	7 (41.2%)
**Total days of ventilator use**	2.5 (1–16)
**Anti-hypertensive use**	1 (5.9%)
**Anti-epileptic use**	5 (29.4%)
**Opiate use**	8 (47.1%)
**Benzodiazepine use**	7 (41.5%)
**Neuromuscular blockade use**	5 (29.4%)
**Steroid use**	7 (41.2%)
**Alpha-2 agonist use**	2 (11.8%)

(N = 17)

To validate results from the real-time system, we compared our HRV values to those obtained using Kubios. All HRV values can be found in S1 Data. We found that values for all three measures of HRV were highly correlated. Pearson correlation coefficients for SDNN, pNN50, and RMSSD calculated during the first 24 hours of PICU stay were 0.93, 0.92, and 0.89 respectively, and 0.97, 0.97, and 0.99 respectively for the last 24 hours of PICU stay (p < 0.0001 for all values). Bland-Altman plots were created for each HRV measure for the first and last 24 hours. (S1 Fig) Averaged raw HRV values used to generate Bland-Altman plots as well as means and differences can be found in S1 Table. SDNN in both the first and last 24 hours was higher when calculated from our system compared to Kubios with a mean difference of 9.5 ms in the first 24 hours and 10.4 ms in the last 24 hours with greater differences at higher measurement values. There were smaller differences between values for pNN50 (mean difference was 1.14 in the first 24 hours and 0.08 in the last 24 hours) and RMSSD (mean difference of 2.08 ms in the first 24 hours and 0.11 ms in the last 24 hours).

**Fig 2:**
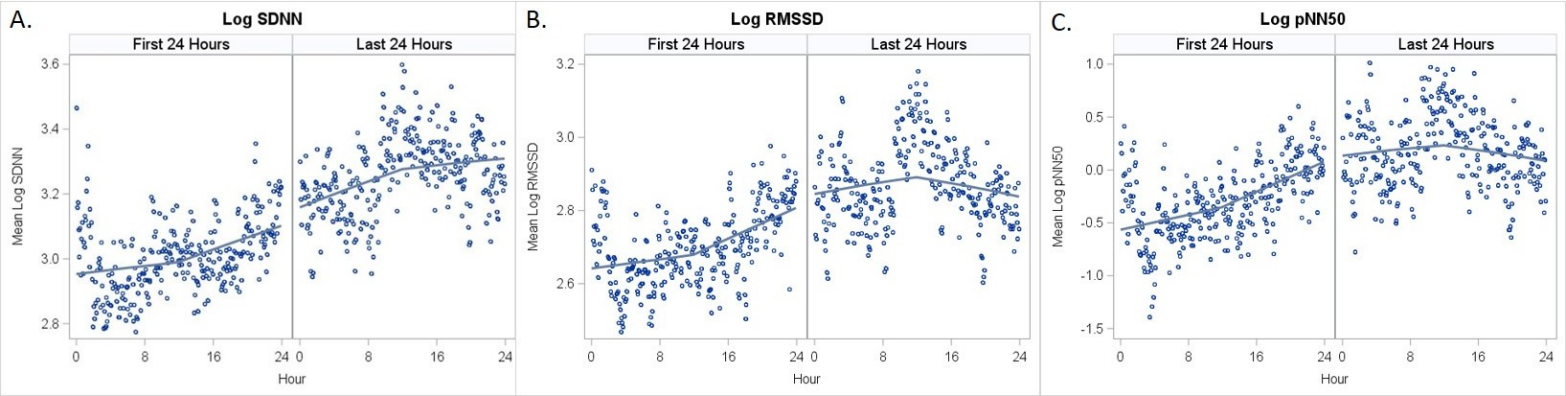
Log heart rate variability over time from PICU admission for first and last 24 hours of PICU stay. Mean HRV for A) SDNN, B) RMSSD, and C) pNN50 displayed from time of PICU admission with HRV from the first 24 hours of PICU stay in the left panel and the last 24 hours in the right panel of each graph. Loess Curves are superimposed.

Visual representations of average HRV measurements are shown in Fig 2. These suggested that there were different relationships in HRV in the first and last 24 hours of admission with HRV appearing higher in the last 24 hours. Mixed effects models for each log-transformed HRV outcome with fixed effects for period of admission indicated statistically significant differences in the first and last 24 hours of PICU stay (Table 2). HRV was higher across all three metrics in the last 24 hours of PICU stay.

**Table 2:** Mixed models evaluating log of the three heart rate variability metrics in the first compared to the last 24 hours of PICU stay.

Outcome	Period	Estimate (95% CI)	P-value
Log SDNN	First 24 Hours Last 24 Hours	-0.21 (-0.23, -0.19) Ref	<0.0001
Log RMSSD	First 24 Hours Last 24 Hours	-0.14 (-0.16, -0.11) Ref	<0.0001
Log pNN50	First 24 Hours Last 24 Hours	-0.41 (-0.48, -0.34) Ref	<0.0001

**Fig 3:**
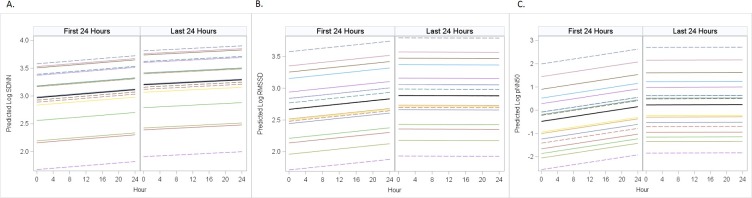
Mixed effects model demonstrating predicted average log heart rate variability in the first vs. last 24 hours of PICU stay. The predicted log HRV is demonstrated for each patient (colored lines) as well as the average value (solid black line) in the first 24 hours of PICU admission (left panel) vs. last 24 hours (right panel). Each colored line indicates an individual patient’s conditional predicted HRV accounting for random effects with the solid black line representing the marginal predicted mean accounting only for fixed effects. Hour represents the number of hours that have passed after admission (left panel) or number of hours that have passed in the 24 hours prior to discharge (right panel). Hour 0 in the first period is the first measurement upon admission; hour 0 in last period is 24 hours before discharge. Mixed models demonstrated that all three measures of HRV were significantly lower during the first 24 hours compared to the last 24 hours of PICU admission (p<0.001).

When evaluating the relationship between time (in hours) from admission and HRV, we found statistically significant interaction terms in log RMSSD and log pNN50 models indicating this relationship was different in the first compared to last 24 hours. (Fig 3) In both models, the slopes of log HRV measures tend to flatten or decrease in the last 24 hours. In the log SDNN model, there was no significant interaction term indicating similar slopes in both the first and last 24 hours. Regardless of significance of interaction terms, the log HRV measures remained consistently higher in the last 24 hours of admission (Fig 3 and Table 2).

We found no significant relationship between demographic or clinical factors (such as diagnosis or medication) and any HRV measurement. However, due to the known association between HRV and age, [25–27] we explored models including age. Adjustment for age did not affect the association between time from admission and log HRV in different time periods (p > 0.1 for all 3 measures).

**Fig 4:**
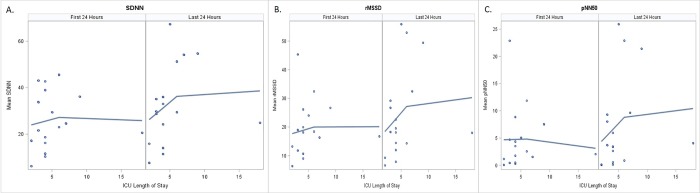
Mean heart rate variability and PICU Length of stay. Mean HRV for a) SDNN; b) RMSSD, and c) pNN50 plotted against PICU length of stay the first 24 hours of PICU stay in the left panel and the last 24 hours in the right panel of each graph.

We also analyzed whether HRV was associated with increasing PICU LOS. HRV in the first 24 hours was not significantly associated with PICU LOS. However, Spearman correlation coefficients showed that mean SDNN (r = 0.52, p = 0.034) and pNN50 (r = 0.51, p = 0.037), but not RMSSD (r = 0.43, p = 0.087), were significantly associated with increasing PICU LOS in the last 24 hours (Fig 4).

## Discussion

The principal finding of this pilot study is that all three measures of HRV were significantly lower in the first 24 hours compared to the last 24 hours of PICU stay. This study also represents the first time, to our knowledge, that HRV in non-neonatal, critically ill pediatric patients was calculated continuously in real-time using an automated streaming analytics platform. In addition to establishing the feasibility of prospective real-time HRV assessment, our results also indicate that greater mean SDNN and pNN50 in the last 24 hours were significantly associated with greater PICU LOS. Taken together, these results suggest that HRV may be a useful marker of clinical acuity in this population though validation in a larger cohort of patients is needed.

Our data add to the body of evidence showing that relatively decreased HRV occurs in the setting of critical illness (such as at the time of PICU admission) and highlight the potential benefit of incorporating real-time HRV analysis into the PICU in the future. Much of the literature on HRV to date focuses on prediction of morbidity or mortality in patients with a specific disease or diagnosis, rather than evaluating its ability to serve as a real-time, overall assessment of clinical acuity. [11,15,28–31] Few studies evaluate the critically ill population as a whole, and even fewer focus on pediatric patients. One notable exception is a group that performed real-time analysis of a type of HRV, called heart rate characteristic (HRC), in infants admitted to two NICUs and assessed risk of sepsis, urinary tract infection, or death. [18] Their results indicated that infants in the NICU with high-risk HRC measurements had a 5 to 6-fold increase in risk of sepsis, UTI, or death in the subsequent week. Their follow-up randomized controlled trial demonstrated a reduction in mortality in very low birthweight infants through continuous HRC monitoring. [20] This led to the development of a commercially available technology, but it is limited to the neonatal population. No equivalent real-time, physiologic assessment tool exists for critically-ill pediatric patients.

The utility of HRV as a marker of clinical acuity is dependent on the ability to accurately measure meaningful changes in real-time. While this study analyzed data retrospectively, all HRV data were calculated prospectively in real-time and stored for analysis. These values also correlated significantly with HRV values obtained from Kubios, a validated software program that requires time to manually load raw RR-intervals for retrospective calculation. Additionally, values from the real-time system demonstrated agreement with those from Kubios with the exception of SDNN, which was consistently higher when calculated using the real-time system with increasing discrepancy as HRV values increased. This could be secondary to more motion artifact or possibly from greater influence of respiratory sinus arrhythmia in healthier children with higher HRV values along with differential management of these phenomena between the two systems since artifact management techniques perform differently based on the HRV metric of interest.[32] Additional evaluation in future studies is necessary. Even with over-estimation of SDNN at higher values, these results indicate that, overall, our system is able to produce comparable results to those produced by validated software.

The results of this pilot study indicate that HRV is lower during the first 24 hours of PICU stay compared to the time period just prior to PICU discharge, supporting its ability to serve as a marker of clinical acuity. This is not surprising considering that ANS dysregulation results from multiple factors including inflammation and metabolic derangements, all of which contribute to organ dysfunction, a hallmark of the critically ill population and often a major contributing factor to PICU admission. [4,5,7,8] We also see evidence of this in the visual representations of mean HRV trended over time from admission and in the day prior to discharge. Here, HRV appears to increase over time in the first 24 hours of admission with overall lowest HRV values appearing to occur within the first several hours of PICU stay. This is consistent with the natural course of critical illness, as the most severe clinical manifestations of critical illness tend to occur in the early part of PICU admission with most patients improving over time with medical interventions.

Our findings that higher HRV in the last 24 hours of PICU stay was associated with increased PICU LOS, but that lower HRV in the first 24 hours was not associated with PICU LOS have not previously been reported. There are several possible explanations for this finding. Children with longer PICU LOS have more time to recover from critical illness while being monitored. It is possible that HRV does not return to baseline values by the time of PICU discharge for all patients. The trajectory of recovery of HRV may depend on disease type and severity. However, these findings suggest that additional evaluation of using HRV to assist in clinical assessment of readiness for PICU discharge may be useful.

There are several limitations to this study. The small sample size and the retrospective nature of the analysis limit conclusions that can be drawn. A significant proportion of patients were excluded from analysis due to incomplete ECG data. This occurred in the setting of inadequate server storage that has since been addressed, but emphasizes the importance of an inter-disciplinary collaboration to assure appropriate infrastructure and technical support.

None of the demographic and clinical variables we evaluated were associated with changes in HRV, potentially due to our limited sample size. While age is known to be associated with HRV in children, our patients were relatively similar in age, potentially reducing our ability to detect this effect. Alternatively, the effects of these variables on HRV may be blunted with critical illness.

Additionally, our real-time system was not designed to adjust for heart rate when calculating HRV, which could significantly alter our findings. Multiple recent studies demonstrated an inverse relationship between HRV and heart rate. [33–36] However, additional factors such as the patient’s physiologic state, sex, and outcome of interest seem to affect whether adjustment for heart rate affects the prognostic capabilities of HRV. Pradhapan et al. demonstrated that accounting for the influence of HR on HRV worsened its prognostic ability when measured in subjects at rest compared to when subjects were recovering from exercise. [35] Further, Sacha et al. found that adjusting for heart rate affected the predictive capability of HRV for cardiac death more in males than females and that heart rate did not contribute to prognostic ability of HRV when the outcome of interest was non-cardiac. [36,37]. They proposed that modifying the dependence of HRV on HR can provide important information about risks for different outcomes. This implies that heart rate may play more of a role in adverse cardiovascular outcomes and that adjusting HRV for heart rate may be appropriate in specific circumstances. However, further evaluation is needed to provide definitive answers.

An additional concern is the presence of artifact which may alter HRV values. We used an automated outlier detection process to eliminate presumptive non-sinus beats. In-person review of electrocardiographic tracings was not done prior to analysis since HRV calculations were performed in real-time. Despite these efforts at artifact management, there likely remain non-sinus beats that were included in our HRV calculations that may alter the final values. However, in order to create a truly functional platform for real-time calculation of HRV we must be able to accommodate some artifact. While intermittent inaccuracies may occur, it is important to determine the significance of the trend over time even with those errors.

In order to further elucidate these findings, future efforts will incorporate analysis of frequency domain and non-linear measures with adjustment for heart rate for a more comprehensive assessment of HRV. In this pilot study, only the first and last 24 hours of HRV were analyzed due to our desire to compare a defined time of critical illness with a defined time of resolution of critical illness using clinician-determined identification of patient status. Our next step is to validate our findings on a larger scale and will include all PICU patients as well as longitudinal data as it is likely that HRV may change throughout ICU stay as patient illness severity changes. Future efforts will also focus on evaluating whether HRV is able to predict changes in clinical status including worsening organ dysfunction.

## Conclusion

This study represents a first step in applying real-time HRV calculation in the PICU and demonstrates that changes in HRV can be detected over time. These findings suggest that HRV analysis may be able to augment the clinician’s understanding of their patient’s physiologic state as it alters between one of critical illness and relative health, which support the hypothesis that HRV would be a useful marker of clinical acuity in patients admitted to the PICU. However, further studies on a larger scale are needed to gain additional insight into the significance of HRV changes in this population.

## Supporting information

S1 Data Patient data and heart rate variability values for all evaluated patients. (XLSX)

S1 Fig Bland-Altman plots for comparison of HRV from our real-time system and Kubios. Bland-Altman plots are shown for the first 24 hours (left panel) and last 24 hours (right panel) of PICU admission for A) SDNN, B) pNN50, and C) RMSSD. The mean differences (represented by the horizontal solid line) for A) were 9.5 and 10.4, B) 1.14 and 0.08, and C) 2.08 and 0.11 for the first and last 24 hours respectively.
(TIF)

S1 Table Raw average HRV values used to in Bland-Altman Plots. The raw HRV values averaged over the first and last 24 hours of PICU stay along with the differences and means are included.
(XLSX)
