# Supplementary figures and images for: Heart rate variability as a marker of recovery from critical illness in children
Source: PLoS One. 2019 May 17;14(5):e0215930. doi: 10.1371/journal.pone.0215930 (PMC6524820; doi:10.1371/journal.pone.0215930)

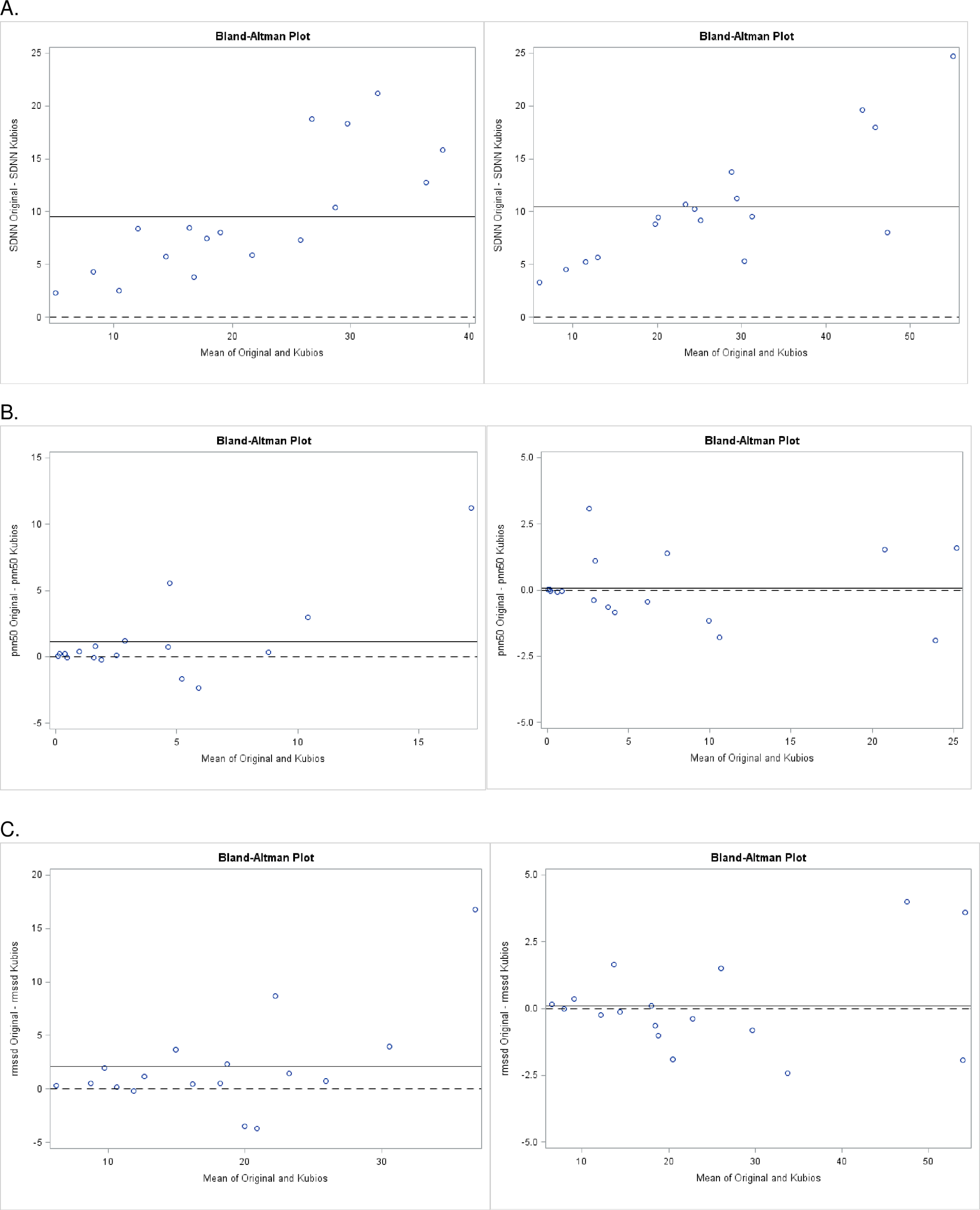

Supplement: S1 Fig — Bland-Altman plots are shown for the first 24 hours (left panel) and last 24 hours (right panel) of PICU admission for A) SDNN, B) pNN50, and C) RMSSD. The mean differences (represented by the horizontal solid line) for A) were 9.5 and 10.4, B) 1.14 and 0.08, and C) 2.08 and 0.11 for the first and last 24 hours respectively. (TIF) [file pone.0215930.s002.tif]
